# Identification of bioactive compounds and inhibitory effects of TNF‐α and COX‐2 in the extract from cultured three‐spot seahorse (*H. trimaculatus*)

**DOI:** 10.1002/fsn3.3824

**Published:** 2023-11-27

**Authors:** Yung‐Husan Chen, Yu‐Wei Chang, Chu‐Wen Ma, Lian‐Zhong Luo, Ting‐Jang Lu, Jeng‐Yuan Yao

**Affiliations:** ^1^ Xiamen Key Laboratory of Marine Medicinal Natural Products Resources Xiamen Medical College Xiamen China; ^2^ Fujian Provincial University Marine Biomedical Resources Engineering Research center Xiamen Medical College Xiamen China; ^3^ Department of Food Science National Taiwan Ocean University Keelung City Taiwan; ^4^ Graduate Institute of Food Science and Technology National Taiwan University Taipei Taiwan; ^5^ Xiamen Key Laboratory of Marine Medicinal Natural Products and Cell Engineering Xiamen Medical College Xiamen China; ^6^ Key Laboratory of Functional and Clinical Translational Medicine Fujian Province University, Xiamen Medical College Xiamen China

**Keywords:** arginine, HPLC‐ESI/MS/MS, taurine, three‐spot seahorse

## Abstract

Three‐spot seahorse (*Hippocampus trimaculatus*) has been consumed as traditional Chinese medicine in Asian society. This study was designed to analyze the bioactive compounds of the solvent extracts from cultured three‐spot seahorse by high pressure liquid chromatography coupled with electrospray ionization tandem mass spectrometry (HPLC‐ESI/MS/MS). Subsequently, their biological activities were evaluated and confirmed by cell modes and Western blot analysis. Experimental results indicated that taurine and arginine were the primary bioactive compounds identified and quantified without pre‐ or post‐column derivatization within 20 min retention time. The analytical method was established and validated with intraday/interday RSD from 0.25% to 3.34% and with recovery from 87.8% to 91.2%. As compared to other extracts, water layer extract (WLE) contained the most taurine and arginine contents of 6.807 and 0.437 mg/g (dry basis), respectively. In the meanwhile, WLE also showed anti‐inflammatory activity on LPS‐induced NO production and inhibited the protein expression of TNF‐α and COX‐2 by Western blot analysis with better cell viability.

## Graphical abstract

The HPLC‐ESI/MS/MS was used to analyze the bioactive components of various solvent extracts from cultured hippocampus trimaculatus. Taurine and arginine were found in the extracts. The cell test and inflammatory analysis supported that the WLE was the better extract that can inhibit LPS‐induced cell production of nitric oxide, and inhibit the expression of inflammatory factors such as COX‐2 and TNF‐α.

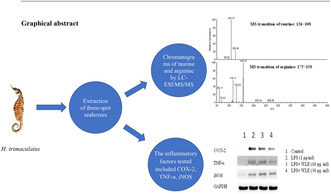

## 1 INTRODUCTION

Traditionally dry three‐spot seahorse (*Hippocampus trimaculatus*) has been consumed in Asian society as a kind of Chinese medicine. It is sweet in taste and gently considered warm to human body. The Chinese Pharmacopeia stipulates that the dried body of three‐spot seahorse (*H. trimaculatus*) can be used as medicine and studies suggest that seahorses have ethnopharmacological characteristics, such as fertility, antioxidants, and anti‐fatigue (Liao et al., 2017; Mundijo, Midoen, et al., 2022; Mundijo, Suyatna, et al., 2022; Jiang et al., 2014). In the “Compendium of Materia Medica,” it is recorded that the seahorse has aphrodisiac effect (Xu et al., 2003). In the meanwhile, some scholars have found that the seahorse can increase the level of serum testosterone (Kim et al., 2016) and has an androgen effect (Zhang, 2004). Additionally, a certain effect on the treatment of male infertility was also confirmed. In recent years, pharmacological research showed that seahorse not only has hormone‐like effect enhancing hematopoietic function, but also shows anti‐cancer, anti‐aging, anti‐fatigue, and Ca^2+^ blocking effects (Chen et al., 2017). It is also been found that after 12 weeks of continuous intake of seahorse enzymatic hydrolysate in male mice, the sperm motility and sperm count of male mice increased, and the level of testosterone increased. This effect mainly due to alkaline protease hydrolysate (ALC) and pepsin hydrolysate (PEP) stimulating the proliferation of mouse Leydig cells (testosterone hormone is mainly secreted and proliferated by these cells), which increases serum testosterone levels. As a result, the diverse pharmacological activities of the seahorse make it a research hotspot recently (Mundijo et al., 2023; Mundijo, Midoen, et al., 2022; Mundijo, Suyatna, et al., 2022).

Taurine and arginine play a very important role in the active function of the human body (Siregar et al., 2022; Xu et al., 2023). For example, taurine has the functions of combining with bile acid to form bile salts and then absorbing the fat, anti‐oxidation, and detoxification. (Chen et al., 2015a, 2015b). Arginine is the precursor of nitric oxide (NO) synthesis in the human body (Kim et al., 2020; Loscalzo., 2000). Nitric oxide synthase (iNOS) can use the nitrogen source and oxygen of arginine to promote the synthesis of NO, thereby promoting vasodilation and blood circulation, while reducing the risk of cardiovascular disease (Wu et al., 2017). It is known that taurine and arginine are active ingredients contained in the seahorse. Detecting the content of taurine and arginine in hippocampus is helpful for species identification, quality evaluation, and pharma‐dynamic activity detection of seahorse (Schuller‐Levis & Eunkyue, 2003; Spitze et al., 2010). However, the existing detection methods for taurine, arginine often require pre‐column derivatization or post‐column derivatization by HPLC, and the operation is tedious. LC‐ESI/MS/MS is widely used in the detection of small organic molecules such as amino acids (Tshepho et al., 2023; Wu et al., 2020; Chen et al., 2015a, 2015b; Chaimbault et al., 2004). The advantage of this method requires less amount of the sample, with the characteristics of detection specificity and high sensitivity (Mahdhi et al., 2021). In addition, for most of the compounds that are not absorbed by UV, they can be detected by mass spectrometers using appropriate methods. However, there are few reports on the detection of taurine and arginine in three‐spotted seahorse (*H. trimaculatus*) by LC‐ESI/MS/MS method. In this study, cultured three‐spotted seahorses (*H. trimaculatus*) were extracted by different solvents. The extracts were further analyzed by the determination of taurine and arginine contents using LC‐ESI/MS/MS approach accordingly. In addition, the cell mode and the anti‐inflammatory assay inhibiting the protein expression of inflammatory factors such as TNF‐α and COX‐2 of the extracts were also examined for evaluating their physiological activities.

## 2 MATERIALS AND METHODS

### 2.1 Materials

Three‐spot seahorse (*H. trimaculatus*) was collected from Danzhou, Hainan, China, in September 2018, and the identity of it was confirmed by Dr. Dexiang Wang (State Key Laboratory of Marine Environmental Science, Xiamen University). *H. trimaculatus* was stored under −20°C in the Marine bio‐pharmaceutical resources engineering research center of Xiamen Medical College (Fujian, China), Numbers for Ht‐2018‐2‐5. L‐arginine and taurine were purchased from Sigma‐Aldrich. All chemicals used in this research were of analytical grade.

### 2.2 Extraction of three‐spot seahorses (*H. Trimaculatus*)

**FIGURE 1:**
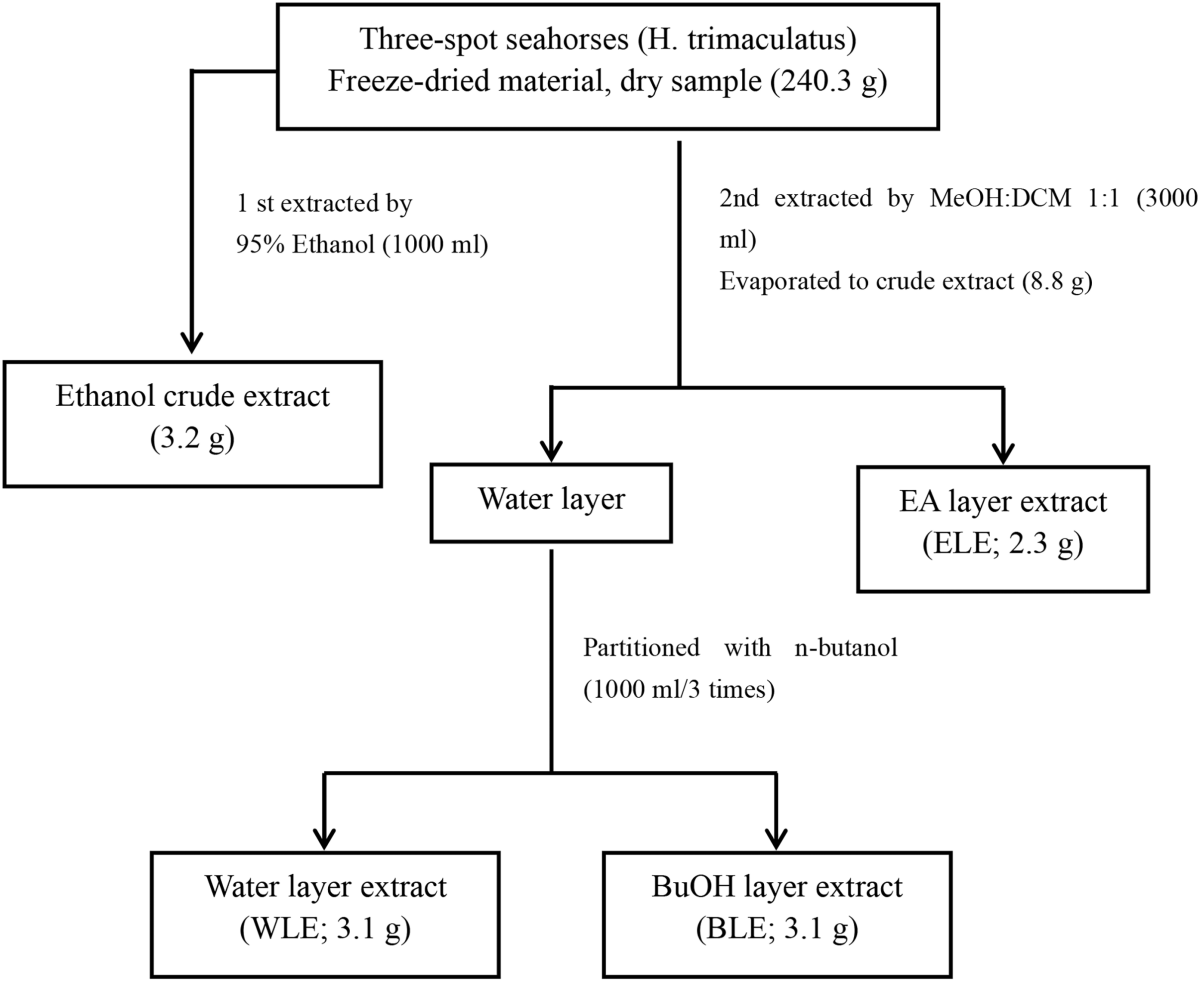
Flow chart of extraction of three‐spot seahorses (*H. trimaculatus*).

As shown in Figure 1, three‐spot seahorses (*H. trimaculatus*) (942.2 g; wet basis) were freeze‐dried (240.3 g; dry basis) and then first extracted with 1000 mL of 95% ethanol to obtain an alcohol extract (ethanol crude extract; 3.2 g). The remaining *H. trimaculatus* was extracted with dichloromethane: methanol (1:1; 3000 mL). After continuously extracting three times, its extract was collected and concentrated using rotary concentrator with reduced pressure. The extracts were further carried out liquid–liquid phase partition extraction three times with extraction of water layer and ethyl acetate and collected ethyl acetate and water extracts respectively. After concentration, the ethyl acetate extract was obtained as EA layer extract (ELE; 2.3 g). The high‐polarity water layer was further divided and extracted three times with n‐butanol. The n‐butanol extracts were combined and concentrated as the BLE (BLE; 3.4 g). Lastly, the remaining WLE was then concentrated under reduced pressure to obtain WLE (WLE; 3.1 g) (Chen et al., 2019).

## 3 LC‐ESI‐MS/MS ANALYSIS OF THREE‐SPOT SEAHORSE EXTRACTS

### 3.1 Sample extraction of free taurine and arginine

Approximately 4 mg of three‐spot seahorse extracts and 20 mg of dispersive‐solid phase extraction (SPE) agent, primary secondary amine (PSA) purchased from Agilent Technologies (New Castle, DE, USA) were mixed with 1 mL of extraction solution (0.1 N HCl, 60% ACN); the aliquot was incubated at 45°C in a dry hot bath (Major Science) with intermittent stir for 15 min then filtered to a brown vial for LC‐ESI tandem MS injection.

### 3.2 Construction of standard calibration curves & method validation

External standard calibration curves were established from a standard mix of taurine and arginine standard solutions (0.1 N HCl, 60% ACN) at 6 concentrations Method accuracy and precision, which were evaluated by adding the spiked standard mix (concentration at 25 μM) into the matrix of seahorse extracts with three measurements, were expressed as recovery (%) and relative standard deviation (RSD) (%), respectively.

### 3.3 LC‐ESI‐MS/MS analysis

One μL of taurine and arginine content was inject into the LC‐ESI‐MS/MS system (LC system: Waters CapLC; MS system: Waters Micromass QTOF Ultima Global, Micromass). The flow rate of the LC system was 6 μL/min through the column system (Waters Atlantis dC_18_”, 3 μm, 75 μm × 50 μm column) and the flow rate eventually was reduced to 0.3 μL/min by the splitter before entering into chromatographic column system. The solvent gradient system consisted of solvent A (H_2_O with 0.1% nonafluorovaleric acid (NFPA)) and solvent B (acetonitrile (ACN) with 0.1% nonafluorovaleric acid (NFPA)). The solvent B increased from 5% to 60% in 25 minutes, then dropped back to 5% in 5 minutes and then equilibrated for 5 minutes. Mass spectrometry was performed by +ESI ionization mode with voltage 4.50 kV and desolvation temperature 290°C. Time‐of‐flight detection was controlled at an accelerating voltage of 9.1 kV, cone voltage of 100 V, and impact energy of 10 eV. The scanning range of mass spectrometry was monitored in between *m/z* 50–500. The selected reaction model (SRM) mode was used to quantify the taurine and arginine contents. The ion chromatography peak area of the product ion was compared with the standard, and then the contents of taurine and arginine in the three‐spotted seahorse (*H. trimaculatus*) extracts were calculated. The mass spectrometer tuning and calibration standard was [Glu]‐Fibrinopeptide B (Sigma Chemicals), and the software for instrument control and data collection were MassLynx V4.0 and ProteinLynx Global Server 2.1 software packages (Suliburska et al., 2014; Thiele et al., 2008).

## 4 ANTI‐INFLAMMATORY ACTIVITIES OF THREE‐SPOT SEAHORSE EXTRACTS ON LPS‐INDUCED CHANGES IN RAW 264.7 CELLS

### 4.1 Cell culture

RAW 264.7, the mouse macrophage cell lines, were obtained from the Food Industry Research and Development Institute, China. The cells were cultured in DMEM medium supplemented with 10% fetal bovine serum in a humidified incubator at 37°C under 5% CO_2_/95% air. Sub‐cultivation was performed 2–3 times per week when the cells were grown to 70%–80% confluence.

### 4.2 Determination of NO concentration and cell viability

RAW264.7 were cultured in 96‐well plates overnight (5000 cells per well). Afterward, LPS (1 μM) was added with/without the addition and incubated with different extracts for 24 h. The NO concentration of the cell culture medium was measured according to the manual of the kit (Beyotime). The measurement of cell viability was performed according to the manual of the kit (GlpBio). RAW 264.7 cells were cultured to 70%–80% confluence to measure cell viability and proliferation with CCK‐8 kit (Abcam). Assay protocols were performed according to the user's manual.

### 4.3 Western blot analysis

RAW 264.7 cells (5 × 106/dish) were cultured in a 100‐mm dish. After incubation overnight, the cells were co‐incubated with LPS (0.5 μg/mL) and analytes for 24 h. Subsequently, the cells were washed twice with ice‐cold PBS and lysed in Mammalian Protein Extraction Reagent (M‐PER; Pierce). After centrifugation, the supernatant was collected, and the protein concentration of the lysate was determined by a BCA protein assay kit (Pierece). The protein samples (10 μg) were fractionated on a 10% SDS polyacrylamide gel and blot‐ted onto polyvinylidene fluoride membranes (Millipore). The membranes were probed with primary antibodies (iNOS, COX2 and GAPDH) at 1:1000 dilutions and secondary antibodies at 1:10,000 dilutions. Immunoreactive protein bands were visualized by Enhanced Chemiluminescence (Amersham Biosciences). For the control group, cell cultures were incubated with the same culture medium of equal volume.

## 5 STATISTICAL ANALYSIS

Analytical determinations for the samples were conducted in triplicate or sextuplicate as mean and relative standard deviations or standard deviations. Student's *t*‐test was used for comparisons between various treatment groups and LPS control group. A *p*‐value less than 0.05 was considered statistically significant. All data were analyzed with InStat3 or Prism software 5.0b (GraphPad Software).

## 6 RESULTS AND DISCUSSION

### 6.1 Determination of taurine and arginine contents of three‐spot seahorse extracts by LC‐ESI‐MS/MS analysis

**FIGURE 2:**
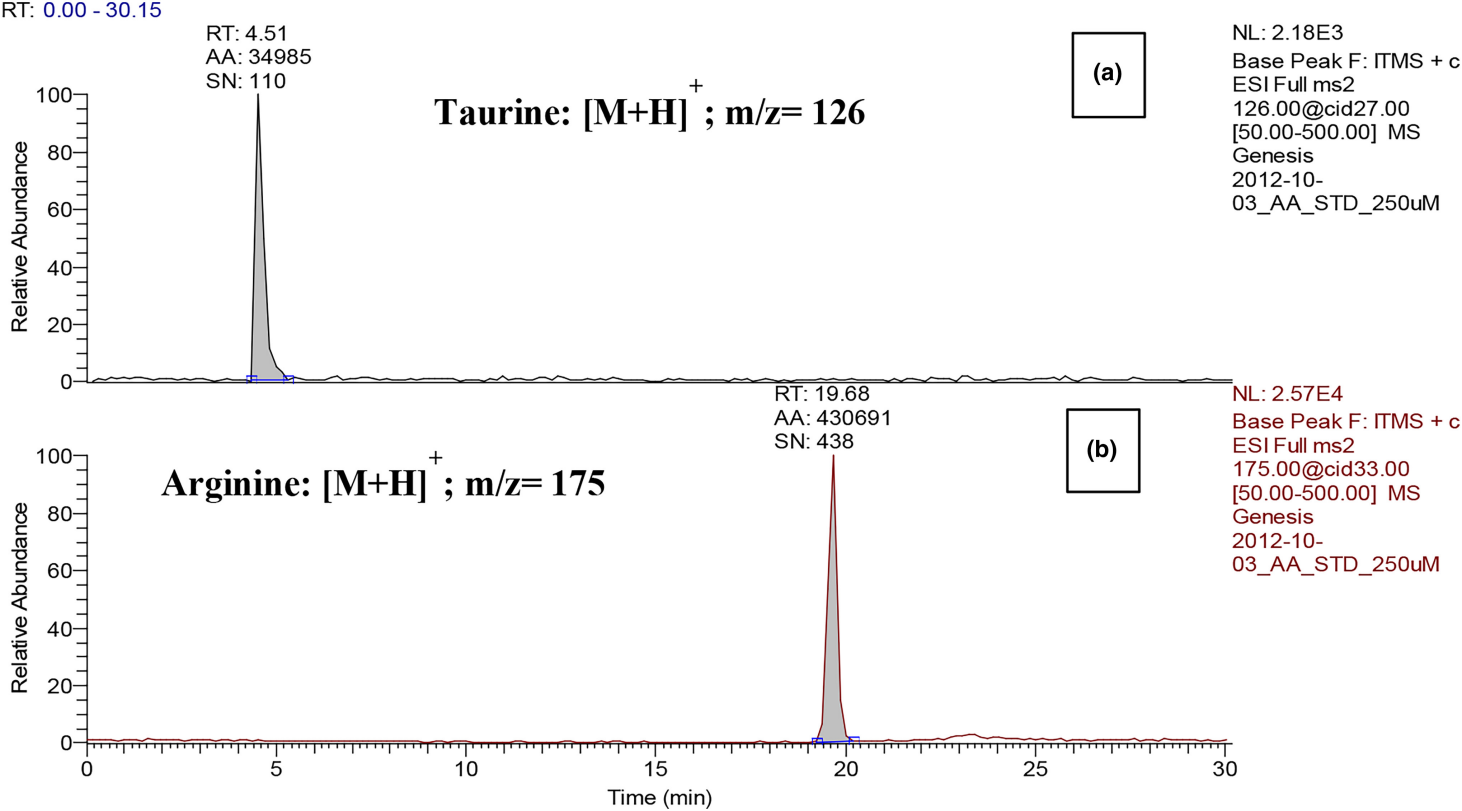
Ion chromatograms of taurine and arginine by LC‐ESI/MS/MS analysis.

**FIGURE 3:**
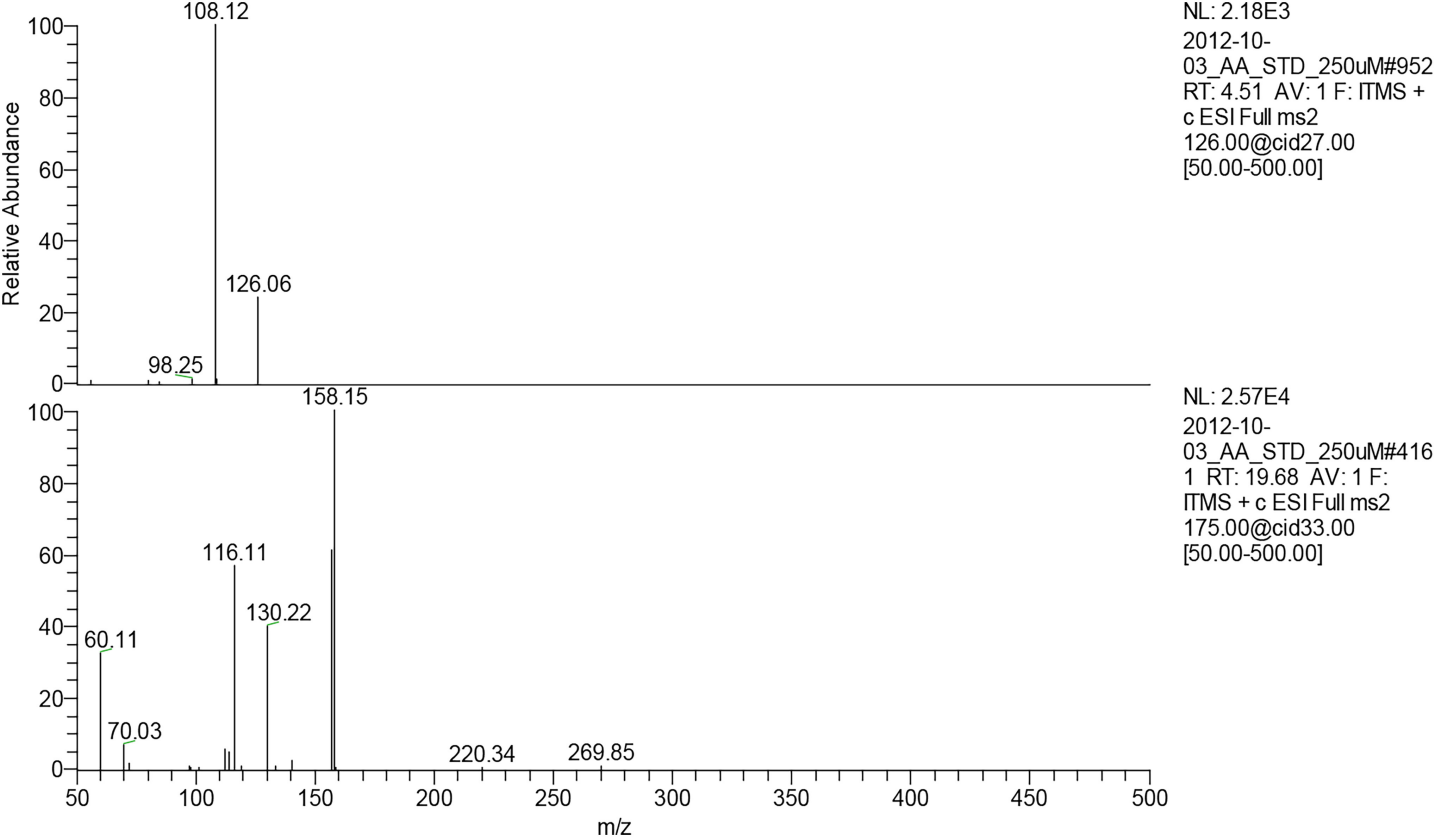
Ion spectra of production ions (MS2) of taurine and arginine by LC‐ESI/MS/MS analysis.

Taurine and arginine serve important biological functions of the human body as mentioned before. In this study, three‐spot seahorse (*H. trimaculatus*) extracts were obtained by the sequential extraction as shown in Figure 1. The three‐spot seahorse extracts, crude extract (CE), EA layer extract (ELE), water layer extract (WLE), and BuOH layer extract (BLE) were determined using taurine and arginine as the indicative components for their physiological activity. In recent years, LC‐ESI/MS/MS method has been widely used in the detection of small organic compounds such as amino acids (Chen et al., 2015a, 2015b; Wu et al., 2017). The advantages of this method are that it requires less sample amount, has the characteristics of detection specificity and high sensitivity, and is also suitable for most compounds that are not absorbed by UV. Therefore, taurine and arginine are ideal to be detected by a mass spectrometer using an appropriate dissociation method. A set of special LC‐ESI/MS/MS analysis method can detect the content of taurine and arginine without going through pre‐ or post‐derivatization HPLC method. As displayed in Figure 2, the retention time (Rt) of taurine and arginine in ion chromatogram were 4.51 and 19.68 min, respectively whereas the *m/z* ratio of taurine and arginine were 126 [M + H]^+^ and 175 [M + H]^+^, respectively. Figure 3 showed the product ion spectra of taurine and arginine giving the ion transition of *m/z* ratio of 126 > 108 and 175 > 158, respectively; the amounts of taurine and arginine were calculated on the basis of selected reaction model (SRM) using the peak area in relation to the taurine and arginine standard calibration curves. In Table 1, the optimized parameters of collision energies for taurine and arginine determined to produce relatively abundant ions were 27 and 22 eV, respectively. In Table 2, the limits of detection (LOD) of taurine and arginine were 0.313 and 0.174 μg/mL, respectively, whereas the limits of quantification (LOQ) were 0.625 and 0.435 μg/mL, respectively. As for the consideration of method validation shown in Table 3, this developed method provided good intraday and interday precisions ranging from 0.25% to 3.34% within comparisons of retention time (Rt) and peak area from ion chromatograms (Pk). In addition, the recovery values of spiked standards (taurine and arginine) were close to 90%.

**TABLE 1:** Optimized MS parameters for SRM determination of underivatized Taurine and L‐arginine.

Analyte	Molecular ion [M + H]^+^, (*m/z*)	Product ion (*m/z*)	Collision energy (eV)
Taurine (Tau)	126	108	27
L‐arginine (Arg)	175	158	33

**TABLE 2:** Method performance parameters.

Analyte	*R* ^2^	Equations	LOD (μg/mL)^a^	LOQ (μg/mL)^b^
Tau	.9961	*y* = 207.3x − 5468.8	0.313	0.625
Arg	.9979	*y* = 3745.6x +602.53	0.174	0.435

^a^ Limit of detection (LOD) was defined as the concentration of each analyte corresponded to a signal‐to‐noise (S/N) ratio approximately equals to 3.

^b^ Limit of quantification (LOQ) was defined as the concentration of each analyte corresponded to a signal‐to‐noise (S/N) ratio approximately equals to 10.

**TABLE 3:** Method validation data.

Analyte	Precision (%) (*n* = 3)				Recovery (%) (*n* = 3)	
	Intraday RSD		Interday RSD			
	Rt^a^	Pk^b^	Rt^a^	Pk^b^	Mean	RSD
Tau	0.25	0.61	1.14	3.19	87.8	1.1
Arg	0.45	2.74	0.80	3.34	91.2	3.3

^a^ Refers to retention time.

^b^ Refers to peak area from ion chromatograms.

The measured contents of taurine and arginine of three‐spot seahorses (*H. trimaculatus*) are displayed in Table 4. Taurine was not detected in CE but arginine was present with trace amount (0.006 mg/g; dry basis). Both taurine and arginine were not found in EAE. On the other hand, they were detected in more hydrophilic layers, BLE and WLE, especially the WLE contained most abundance of taurine and arginine of 6.807 and 0.437 mg/g (dry basis), respectively. The total amount of combing the contents of taurine from BLE and WLE was 8.454 mg/g (dry basis). In comparison, this amount is actually higher than most of taurine contents from animal sources such as beef (0.4–8.1 mg/g), pork (2.4–3.3 mg/g), chicken (0.5–7.3 mg/g), and seafood (0.9–9.2 mg/g) based on dry weight basis (Spitze et al., 2010). This result indicates that the extraction process is suitable to isolate taurine and arginine from three‐spot seahorses (*H. trimaculatus*) in particular the WLE.

**TABLE 4:** Results of taurine and arginine content.

Analyte	Seashore extracts (mg/g; dry basis) (*n* = 3)			
	CE*	ELE*	BLE*	WLE*
	Mean (RSD%)	Mean (RSD%)	Mean (RSD%)	Mean (RSD%)
Tau	<LOD	<LOD	1.647 (5.9)	6.807 (8.4)
Arg	0.006 (1.8)	<LOD	0.037 (4.6)	0.437 (2.6)

* BLE, BuOH layer extract; CE, Crude extract; EAE, EA layer extract; WLE, water layer extract.

### 6.2 Anti‐inflammatory activity of three‐spot seahorse (*H. Trimaculatus*) extracts on LPS‐induced changes in RAW 264.7 cells

**FIGURE 4:**
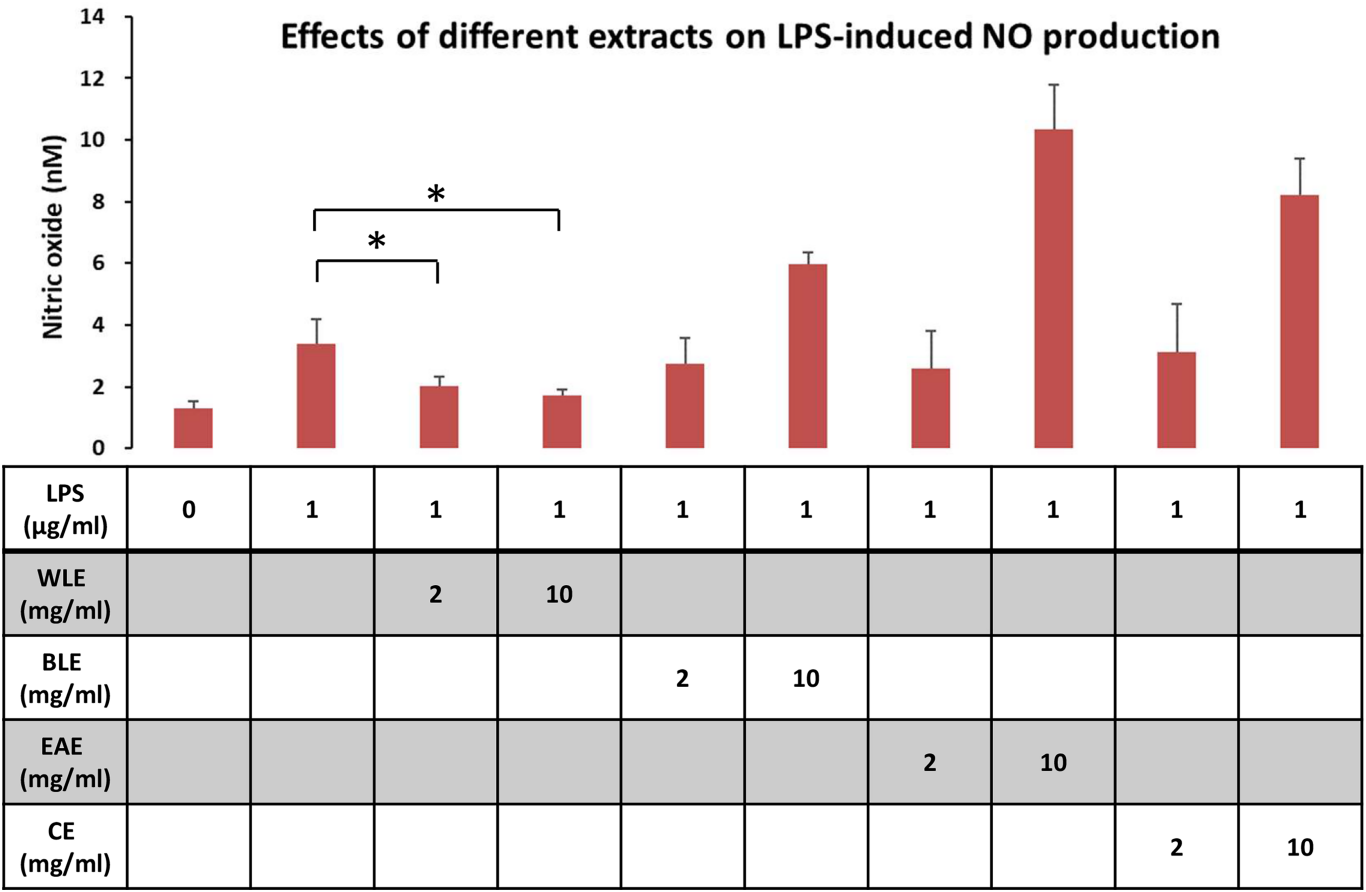
Inhibition of LPS‐induced NO production by extracts of three‐spot seahorse *(H. trimaculatus*). The impact of various extracts on the elevation of NO levels induced by LPS was investigated using RAW264.7 cells. Each extract's effect was assessed independently. The data is represented as the mean ± standard deviation (S.D.) from six distinct experiments. Statistical significance was determined through a *t*‐test; significance is denoted by an asterisk (*), indicating a *p*‐value of ≤0.05.

The experimental results showed that LPS treatment significantly increased the NO concentration in the cell culture medium as compared to the control group in Figure 4. LPS + WLE (2 mg/mL) group significantly reduced the LPS‐induced increase in NO concentration. Particularly with LPS + WLE (10 mg/mL) group, the NO production decreased even more but not significant. EAE, BLE, and CE treat did not reduce NO concentration, and even increased NO concentration at high concentrations. NO is an important reactive molecule that plays an important role in intercellular and intracellular signaling and is involved in mediating immune and inflammatory responses (Wu et al., 2013). In this article, the commonly used Griess method was performed to measure the NO_2_ content of cell culture supernatant, which indirectly reflected the NO production of macrophages. Treatment of WLE reduced LPS‐induced NO production in a concentration‐dependent manner. Extracts from the other three layers were not able to reduce LPS‐induced NO production and induced more NO production at high concentrations.

### 6.3 Determination of NO concentration and cell viability

**FIGURE 5:**
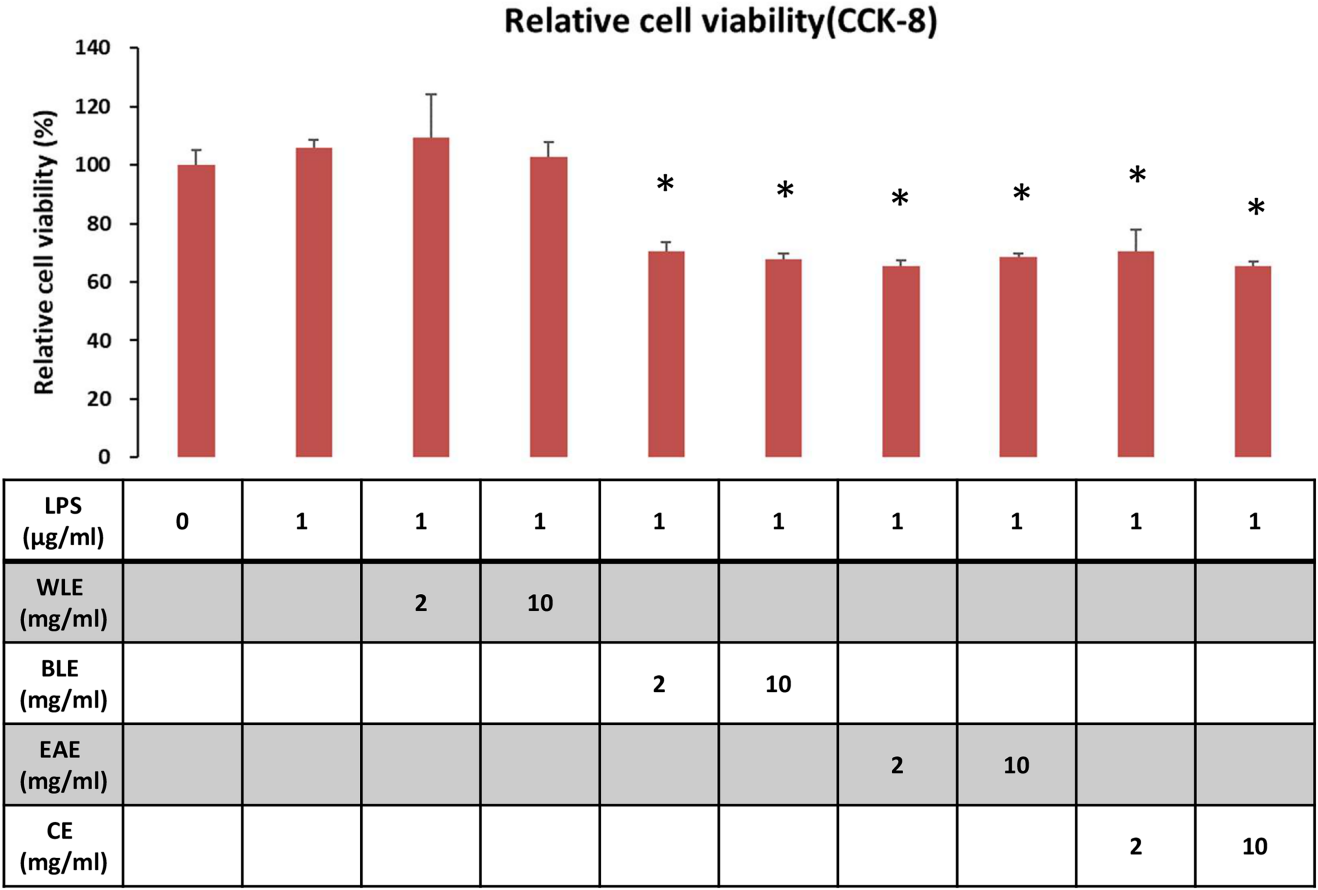
Assessment of relative cell viability using CCK‐8 assay for experimental groups. Figure 5. Assessment of relative cell viability using CCK‐8 assay for experimental groups from Figure 4. Each extract's effect was assessed independently. The data is represented as the mean ± standard deviation (S.D.) from six distinct experiments. Statistical significance was determined through a *t*‐test; significance is denoted by an asterisk (*), indicating a *p*‐value of ≤0.05.

In Figure 5, the cell viability analysis showed that there was no significant difference between the LPS and LPS + WLE treat groups and the control group. However, the LPS + EAE, LPS + BLE, and LPS + CE groups significantly decreased cell viability. The ability to promote cell proliferation is an important indicator to identify the quality of the medium, and its detection method is also crucial. Such as the method of rapid cell proliferation detection by CCK‐8 staining, this study analyzed the factors that affect the results of cell proliferation detection through a series of experiments. Using the CCK‐8 method, it was found that CE, BLE, and ELE significantly reduced cell viability, but WLE did not affect cell viability. Consequently, WLE seems to be possess great potential without toxic property.

### 6.4 Inflammatory evaluation of WLE by Western blot analysis

**FIGURE 6:**
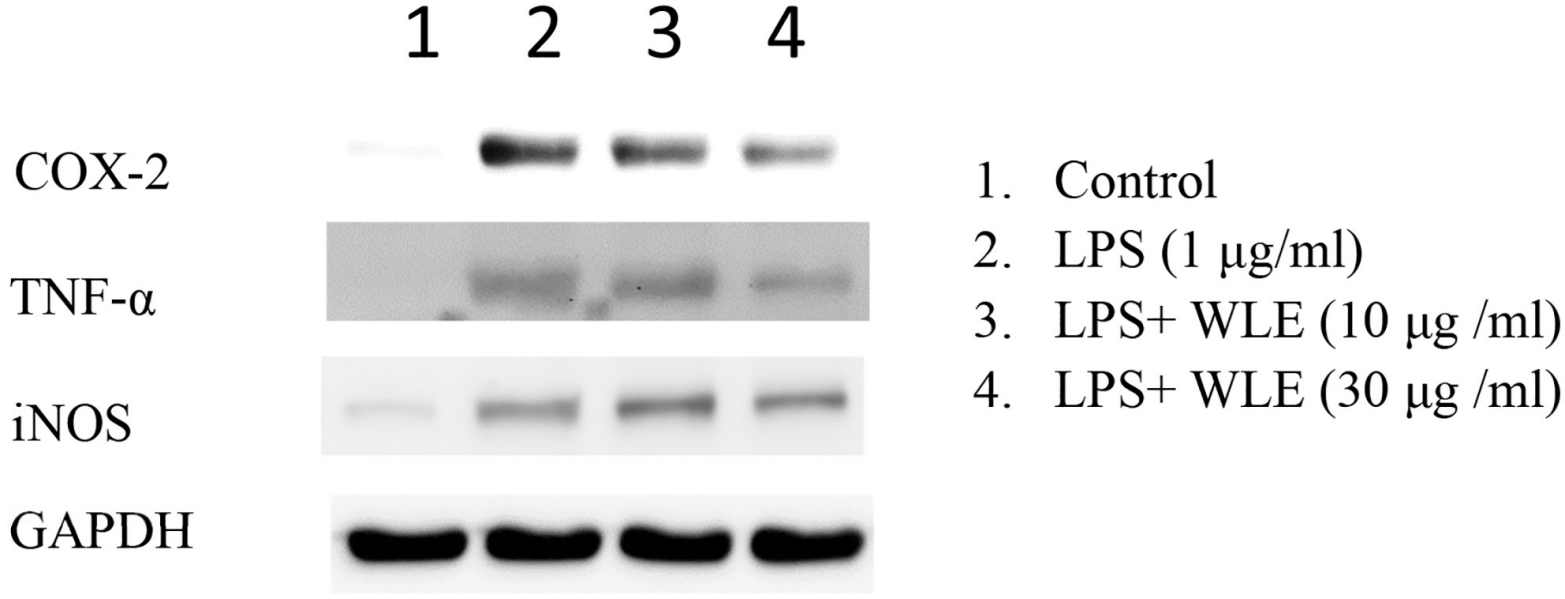
Effects of WLE treatment on LPS‐induced production of inflammatory factors. The effects of low and high concentrations of WLE on the levels of inflammatory factors were tested by WB when LPS‐induced inflammation. The inflammatory factors tested included COX‐2, TNF‐α, and iNOS, and the GAPDH was used as an internal control.

TNF‐α is an important inflammatory mediator, mainly from mononuclear macrophages, and its concentration reflects the intensity of the body's inflammatory response and has a significant correlation with organ damage. Therefore, in this study, the macrophage cell line RAW264.7 was used as the experimental vector to explore the mechanism of extracellular histones mediating the secretion of TNF‐α by macrophages. Concentration dependence wise, TNF‐α has the strongest anti‐tumor activity among the inflammatory cytokines found so far and plays an important role in the occurrence and development of inflammation and tumors. It can promote the production or expression of IL‐6, IL‐ 1β, PGE2, collagenase, and adhesion molecules, etc., further expand the systemic inflammatory response (Heo et al., 2010) Under normal conditions, macrophages hardly express inducible iNOS and inducible cyclooxygenase (COX‐2). Macrophages stimulated by LPS can express a large amount of them. The two are the upstream key enzymes of NO and PGE2 synthesis, which are involved in key steps in the occurrence and development of inflammation, and their regulation is an important target of immune drugs (Xiong et al., 2014; Zhao et al., 2018). As shown in Figure 6, Western blot analysis of WLE on LPS‐induced RAW264.7 cells to produce inflammatory factors indicated that WLE can significantly inhibit LPS‐induced COX‐2 expression in a concentration‐dependent manner. However, WLE had no significant effect on LPS‐induced iNOS production, and both arginine and taurine were reported to regulate TNF‐α expression and have anti‐inflammatory effects (Ahmadian et al., 2017; Suliburska et al., 2014; Sun et al., 2012; Wu et al., 2009).

## 7 CONCLUSIONS

This research had managed to isolate the different solvent extracts from cultured three‐spot seahorses (*H. trimaculatus*) and established a rapid and convenient LC‐ESI/MS/MS method to detect taurine and arginine without derivatization of target compounds. The cell test and inflammatory analysis supported that the WLE was the better extract that can inhibit LPS‐induced cell production of NO and inhibit the expression of inflammatory factors such as COX‐2 and TNF‐α. However, it was found that WLE can reduce the production of NO in cells, it has no significant effect on the expression of iNOS. The mechanism behind remains to be further studied.

## 8 PRACTICAL APPLICATIONS

Three‐spot seahorse (*H. trimaculatus*) can be cultured artificially with massive production. This study has proven WLE from three‐spot seahorse is a great source of taurine and arginine with potential anti‐inflammatory effects. Therefore, WLE can be potential ingredient with increased values for nutraceutical or pharmaceutical industries.

## AUTHOR CONTRIBUTIONS

**Yung‐Husan Chen:** Conceptualization (lead); formal analysis (lead); funding acquisition (equal); methodology (lead); validation (lead); writing – original draft (lead). **Yu‐Wei Chang:** Conceptualization (equal); formal analysis (supporting); funding acquisition (equal); methodology (supporting); validation (equal). **Chu‐Wen Ma:** Validation (supporting); writing – review and editing (lead). **Lian‐Zhong Luo:** Supervision (lead). **Ting‐jang Lu:** Resources (lead). **Jeng‐Yuan Yao:** Conceptualization (equal); funding acquisition (equal); methodology (equal); validation (equal); visualization (equal); writing – original draft (equal).

## FUNDING INFORMATION

This research was supported by Center of Excellence for the Oceans and Ministry of Science and Technology of Taiwan (Grant No: MOST 110‐2320‐B‐019‐007 to Yu‐Wei Chang), Program for New Century Excellent Talents in Fujian Province University (Grant No. NC2018‐47 to Jeng‐Yuan Yao), and 2023 Natural Science Foundation of Fujian Province (Grant No. 2023 J011651 to Yung‐Husan Chen).

## CONFLICT OF INTEREST STATEMENT

The authors hereby declare that there are no conflicts of interest.

## Data Availability

The data that support the findings of this study are available from the corresponding author upon reasonable request.
